# Brown fat protects against hepatic oxidative stress by remodeling the circulating metabolome

**DOI:** 10.64898/2026.05.12.722834

**Published:** 2026-06-10

**Authors:** Dandan Wang, Mark Li, Tian Lu, Mami Matsushita, Juro Sakai, Masayuki Saito, Takeshi Yoneshiro, Shingo Kajimura

## Abstract

Brown adipose tissue (BAT) regulates systemic metabolism beyond thermogenesis, yet the circulating mediators through which BAT communicates with other organs remain less explored. Here, we performed comprehensive serum metabolomics and lipidomics in BAT-ablated mice and human cohorts with varying BAT activity to delineate how BAT activity shapes the circulating metabolome. By integrating datasets across serum, tissues, extracellular fluids, and conditioned media, we assembled BAT-linked circulating molecular signatures. The analyses support a critical role for BAT in the clearance of circulating branched-chain amino acids and triglycerides. We also identified a cold-inducible metabolite, 3-hydroxystearic acid (3-OHSA), produced primarily by BAT and released into circulation. 3-OHSA serves as a circulating readout of cold-activated BAT and acts on the liver to reduce mitochondrial membrane potential and reactive oxygen species production, thereby limiting oxidative stress. This work provides a framework for identifying BAT-derived mediators and uncovers a BAT-liver axis that coordinates adaptation to metabolic stress.

## Full Text Availability

The license terms selected by the author(s) for this preprint version do not permit archiving in PMC. The full text is available from the preprint server.

